# Supplementary material for: Quality of life among patients with cancer and their family caregivers in the Sub-Saharan region: A systematic review of quantitative studies
Source: PLOS Glob Public Health. 2022 Mar 31;2(3):e0000098. doi: 10.1371/journal.pgph.0000098 (PMC10021310; doi:10.1371/journal.pgph.0000098)
Supplement: S1 Appendix — (DOCX) [file pgph.0000098.s002.docx]

**Appendix 1: Search Strategy:**

Database:  PubMed (MEDLINE)

| Set # |  | Results |
| --- | --- | --- |
| 1 | "Palliative Care"[Mesh] OR "Hospice Care"[Mesh] OR "Hospice and Palliative Care Nursing"[Mesh] OR "Holistic Nursing"[Mesh] OR "Holistic Health"[Mesh] OR "Spirituality"[Mesh] OR "Psychosocial Support Systems"[Mesh] OR palliative[tiab] OR “supportive care”[tiab] OR hospice[tiab] OR well-being[tiab] or wellbeing[tiab] OR psychosocial[tiab] OR spirituality[tiab] OR spiritual[tiab] OR spiritualities[tiab] OR holistic[tiab] |  |
| 2 | "Africa South of the Sahara"[Mesh] OR “Sub-Saharan Africa”[tiab] OR “Subsaharan Africa”[tiab] OR “Africa South of the Sahara”[tiab] OR “Central Africa”[tiab] OR “Central African”[tiab] OR "eastern Africa"[tiab] OR "east Africa"[tiab] OR "east African"[tiab] OR “Southern Africa”[tiab] OR “South African”[tiab] OR “West Africa”[tiab] OR “West African”[tiab] OR “Western Africa”[tiab] OR "British Indian Ocean Territory"[tiab] OR Burundi[tiab] OR Umurundi[tiab] OR Abarundi[tiab] OR Burundian[tiab] OR Urundi[tiab] OR Djibouti[tiab] OR Djiboutian[tiab] OR "French Somaliland"[tiab] OR “French Somali”[tiab] OR Eritrea[tiab] OR Eritrean[tiab] OR Ethiopia[tiab] OR Ethiopian[tiab] OR Kenya[tiab] OR Kenyan[tiab] OR Rwanda[tiab] OR Ruanda[tiab] OR Rwandan[tiab] OR Somalia[tiab] OR Somalian[tiab] OR "South Sudan"[tiab] OR Sudan[tiab] OR Sudanese[tiab] OR Tanzania[tiab] OR Tanzanian[tiab] OR Zanzibar[tiab] OR Zanzibari[tiab] OR Tanganyika[tiab] OR Uganda[tiab] OR Ugandan[tiab] OR Cameroon[tiab] OR Cameroonian[tiab] OR “Central African Republic”[tiab] OR Chad[tiab] OR Chadian[tiab] OR Congo[tiab] OR Congolese[tiab] OR “Democratic Republic of the Congo”[tiab] OR “Equatorial Guinea”[tiab] OR “Equatorial Guinean”[tiab] OR Equatoguinean[tiab] OR Gabon[tiab] OR Gabonese[tiab] OR Gabonaise[tiab] OR “Sao Tome and Principe”[tiab] OR “Sao Tomean”[tiab] OR Santomean[tiab] OR Angola[tiab] OR Angolan[tiab] OR Botswana[tiab] OR Botswanan[tiab] OR Motswana[tiab] OR Lesotho[tiab] OR Lesothan[tiab] OR Mosotho[tiab] OR Basotho[tiab] OR Lesothonian[tiab] OR Malawi[tiab] OR Malawian[tiab] OR Mozambique[tiab] OR Mozambican[tiab] OR Namibia[tiab] OR Namibian[tiab] OR “South Africa”[tiab] OR Swaziland[tiab] OR Swazi[tiab] OR Zambia[tiab] OR Zambian[tiab] OR Zimbabwe[tiab] OR Zimbabwean[tiab] OR Benin[tiab] OR Beninese[tiab] OR Beninois[tiab] OR “Burkina Faso”[tiab] OR Burkinese[tiab] OR Burkinabe[tiab] OR “Cabo Verde”[tiab] OR “Cape Verde”[tiab] OR “Cabo Verdean”[tiab] OR “Cape Verdean”[tiab] OR “Cote d'Ivoire”[tiab] OR “Ivory Coast”[tiab] OR Ivorian[tiab] OR Gambia[tiab] OR Gambian[tiab] OR Ghana[tiab] OR Ghanaian[tiab] OR Guinea[tiab] OR Guinean[tiab] OR “Guinea-Bissau”[tiab] OR Bissau-Guinean[tiab] OR Liberia[tiab] OR Liberian[tiab] OR Mali[tiab] OR Malian[tiab] OR Mauritania[tiab] OR Mauritanian[tiab] OR Niger[tiab] OR Nigerian[tiab] OR Nigeria[tiab] OR Senegal[tiab] OR Senegalese[tiab] OR “Sierra Leone”[tiab] OR “Sierra Leonean”[tiab] OR Togo[tiab] OR Togolese[tiab] |  |
| 3 | Neoplasms[Mesh] OR Neoplasms[tiab] OR Neoplasm[tiab] OR Neoplasia[tiab] OR Cancer[tiab] OR Cancers[tiab] OR cancerous[tiab] OR Tumor[tiab] OR Tumors[tiab] OR Tumour[tiab] OR Tumours[tiab] OR carcinoma[tiab] OR carcinomas[tiab] OR Oncology[tiab] OR malignancy[tiab] OR malignancies[tiab] OR malignant[tiab] |  |
| 4 | # 1 AND # 2 AND # 3 | 583 |

Database:  CINAHL Plus with Full Text (EBSCO*host*)

| Set # |  | Results |
| --- | --- | --- |
| 1 | MH "Palliative Care" OR MH "Hospice Care" OR MH "Hospice and Palliative Nursing" OR MH "Holistic Nursing" OR MH "Holistic Health" OR MH "Holistic Care" OR MH "Spirituality" OR MH "Support, Psychosocial+" OR MH "Psychosocial Aspects of Illness" OR TI (palliative OR “supportive care” OR hospice OR well-being OR wellbeing OR psychosocial OR spirituality OR spiritual OR spiritualities OR holistic) OR AB (palliative OR “supportive care” OR hospice OR well-being OR wellbeing OR psychosocial OR spirituality OR spiritual OR spiritualities OR holistic) |  |
| 2 | MH "Africa South of the Sahara+" OR TI (“Sub-Saharan Africa” OR “Subsaharan Africa” OR “Africa South of the Sahara” OR “Central Africa” OR “Central African” OR "eastern Africa" OR "east Africa" OR "east African" OR “Southern Africa” OR “South African” OR “West Africa” OR “West African” OR “Western Africa” OR "British Indian Ocean Territory" OR Burundi OR Umurundi OR Abarundi OR Burundian OR Urundi OR Djibouti OR Djiboutian OR "French Somaliland" OR “French Somali” OR Eritrea OR Eritrean OR Ethiopia OR Ethiopian OR Kenya OR Kenyan OR Rwanda OR Ruanda OR Rwandan OR Somalia OR Somalian OR "South Sudan" OR Sudan OR Sudanese OR Tanzania OR Tanzanian OR Zanzibar OR Zanzibari OR Tanganyika OR Uganda OR Ugandan OR Cameroon OR Cameroonian OR “Central African Republic” OR Chad OR Chadian OR Congo OR Congolese OR “Democratic Republic of the Congo” OR “Equatorial Guinea” OR “Equatorial Guinean” OR Equatoguinean OR Gabon OR Gabonese OR Gabonaise OR “Sao Tome and Principe” OR “Sao Tomean” OR Santomean OR Angola OR Angolan OR Botswana OR Botswanan OR Motswana OR Lesotho OR Lesothan OR Mosotho OR Basotho OR Lesothonian OR Malawi OR Malawian OR Mozambique OR Mozambican OR Namibia OR Namibian OR “South Africa” OR Swaziland OR Swazi OR Zambia OR Zambian OR Zimbabwe OR Zimbabwean OR Benin OR Beninese OR Beninois OR “Burkina Faso” OR Burkinese OR Burkinabe OR “Cabo Verde” OR “Cape Verde” OR “Cabo Verdean” OR “Cape Verdean” OR “Cote d'Ivoire” OR “Ivory Coast” OR Ivorian OR Gambia OR Gambian OR Ghana OR Ghanaian OR Guinea OR Guinean OR “Guinea-Bissau” OR Bissau-Guinean OR Liberia OR Liberian OR Mali OR Malian OR Mauritania OR Mauritanian OR Niger OR Nigerian OR Nigeria OR Senegal OR Senegalese OR “Sierra Leone” OR “Sierra Leonean” OR Togo OR Togolese) OR AB (“Sub-Saharan Africa” OR “Subsaharan Africa” OR “Africa South of the Sahara” OR “Central Africa” OR “Central African” OR "eastern Africa" OR "east Africa" OR "east African" OR “Southern Africa” OR “South African” OR “West Africa” OR “West African” OR “Western Africa” OR "British Indian Ocean Territory" OR Burundi OR Umurundi OR Abarundi OR Burundian OR Urundi OR Djibouti OR Djiboutian OR "French Somaliland" OR “French Somali” OR Eritrea OR Eritrean OR Ethiopia OR Ethiopian OR Kenya OR Kenyan OR Rwanda OR Ruanda OR Rwandan OR Somalia OR Somalian OR "South Sudan" OR Sudan OR Sudanese OR Tanzania OR Tanzanian OR Zanzibar OR Zanzibari OR Tanganyika OR Uganda OR Ugandan OR Cameroon OR Cameroonian OR “Central African Republic” OR Chad OR Chadian OR Congo OR Congolese OR “Democratic Republic of the Congo” OR “Equatorial Guinea” OR “Equatorial Guinean” OR Equatoguinean OR Gabon OR Gabonese OR Gabonaise OR “Sao Tome and Principe” OR “Sao Tomean” OR Santomean OR Angola OR Angolan OR Botswana OR Botswanan OR Motswana OR Lesotho OR Lesothan OR Mosotho OR Basotho OR Lesothonian OR Malawi OR Malawian OR Mozambique OR Mozambican OR Namibia OR Namibian OR “South Africa” OR Swaziland OR Swazi OR Zambia OR Zambian OR Zimbabwe OR Zimbabwean OR Benin OR Beninese OR Beninois OR “Burkina Faso” OR Burkinese OR Burkinabe OR “Cabo Verde” OR “Cape Verde” OR “Cabo Verdean” OR “Cape Verdean” OR “Cote d'Ivoire” OR “Ivory Coast” OR Ivorian OR Gambia OR Gambian OR Ghana OR Ghanaian OR Guinea OR Guinean OR “Guinea-Bissau” OR Bissau-Guinean OR Liberia OR Liberian OR Mali OR Malian OR Mauritania OR Mauritanian OR Niger OR Nigerian OR Nigeria OR Senegal OR Senegalese OR “Sierra Leone” OR “Sierra Leonean” OR Togo OR Togolese) |  |
| 3 | MH "Neoplasms+" OR TI (Neoplasms OR Neoplasm OR Neoplasia OR Cancer OR Cancers OR cancerous OR Tumor OR Tumors OR Tumour OR Tumours OR carcinoma OR carcinomas OR Oncology OR malignancy OR malignancies OR malignant) OR AB (Neoplasms OR Neoplasm OR Neoplasia OR Cancer OR Cancers OR cancerous OR Tumor OR Tumors OR Tumour OR Tumours OR carcinoma OR carcinomas OR Oncology OR malignancy OR malignancies OR malignant) |  |
| 4 | # 1 AND # 2 AND # 3 | 278 |

Database:  Embase (Elsevier)

| Set # |  | Results |
| --- | --- | --- |
| 1 | 'palliative therapy'/exp OR 'hospice care'/exp OR 'palliative nursing'/exp OR 'holistic nursing'/exp OR 'holistic care'/exp OR 'religion'/exp OR 'psychosocial care'/exp OR palliative:ti,ab OR ‘supportive care’:ti,ab OR hospice:ti,ab OR well-being:ti,ab OR wellbeing:ti,ab OR psychosocial:ti,ab OR spirituality:ti,ab OR spiritual:ti,ab OR spiritualities:ti,ab OR holistic:ti,ab |  |
| 2 | 'Africa south of the Sahara'/exp OR ‘Sub-Saharan Africa’:ti,ab OR ‘Subsaharan Africa’:ti,ab OR ‘Africa South of the Sahara’:ti,ab OR ‘Central Africa’:ti,ab OR ‘Central African’:ti,ab OR ‘eastern Africa’:ti,ab OR ‘east Africa’:ti,ab OR ‘east African’:ti,ab OR ‘Southern Africa’:ti,ab OR ‘South African’:ti,ab OR ‘West Africa’:ti,ab OR ‘West African’:ti,ab OR ‘Western Africa’:ti,ab OR ‘British Indian Ocean Territory’:ti,ab OR Burundi:ti,ab OR Umurundi:ti,ab OR Abarundi:ti,ab OR Burundian:ti,ab OR Urundi:ti,ab OR Djibouti:ti,ab OR Djiboutian:ti,ab OR ‘French Somaliland’:ti,ab OR ‘French Somali’:ti,ab OR Eritrea:ti,ab OR Eritrean:ti,ab OR Ethiopia:ti,ab OR Ethiopian:ti,ab OR Kenya:ti,ab OR Kenyan:ti,ab OR Rwanda:ti,ab OR Ruanda:ti,ab OR Rwandan:ti,ab OR Somalia:ti,ab OR Somalian:ti,ab OR ‘South Sudan’:ti,ab OR Sudan:ti,ab OR Sudanese:ti,ab OR Tanzania:ti,ab OR Tanzanian:ti,ab OR Zanzibar:ti,ab OR Zanzibari:ti,ab OR Tanganyika:ti,ab OR Uganda:ti,ab OR Ugandan:ti,ab OR Cameroon:ti,ab OR Cameroonian:ti,ab OR ‘Central African Republic’:ti,ab OR Chad:ti,ab OR Chadian:ti,ab OR Congo:ti,ab OR Congolese:ti,ab OR ‘Democratic Republic of the Congo’:ti,ab OR ‘Equatorial Guinea’:ti,ab OR ‘Equatorial Guinean’:ti,ab OR Equatoguinean:ti,ab OR Gabon:ti,ab OR Gabonese:ti,ab OR Gabonaise:ti,ab OR ‘Sao Tome and Principe’:ti,ab OR ‘Sao Tomean’:ti,ab OR Santomean:ti,ab OR Angola:ti,ab OR Angolan:ti,ab OR Botswana:ti,ab OR Botswanan:ti,ab OR Motswana:ti,ab OR Lesotho:ti,ab OR Lesothan:ti,ab OR Mosotho:ti,ab OR Basotho:ti,ab OR Lesothonian:ti,ab OR Malawi:ti,ab OR Malawian:ti,ab OR Mozambique:ti,ab OR Mozambican:ti,ab OR Namibia:ti,ab OR Namibian:ti,ab OR ‘South Africa’:ti,ab OR Swaziland:ti,ab OR Swazi:ti,ab OR Zambia:ti,ab OR Zambian:ti,ab OR Zimbabwe:ti,ab OR Zimbabwean:ti,ab OR Benin:ti,ab OR Beninese:ti,ab OR Beninois:ti,ab OR ‘Burkina Faso’:ti,ab OR Burkinese:ti,ab OR Burkinabe:ti,ab OR ‘Cabo Verde’:ti,ab OR ‘Cape Verde’:ti,ab OR ‘Cabo Verdean’:ti,ab OR ‘Cape Verdean’:ti,ab OR ‘Cote dIvoire’:ti,ab OR ‘Ivory Coast’:ti,ab OR Ivorian:ti,ab OR Gambia:ti,ab OR Gambian:ti,ab OR Ghana:ti,ab OR Ghanaian:ti,ab OR Guinea:ti,ab OR Guinean:ti,ab OR ‘Guinea-Bissau’:ti,ab OR Bissau-Guinean:ti,ab OR Liberia:ti,ab OR Liberian:ti,ab OR Mali:ti,ab OR Malian:ti,ab OR Mauritania:ti,ab OR Mauritanian:ti,ab OR Niger:ti,ab OR Nigerian:ti,ab OR Nigeria:ti,ab OR Senegal:ti,ab OR Senegalese:ti,ab OR ‘Sierra Leone’:ti,ab OR ‘Sierra Leonean’:ti,ab OR Togo:ti,ab OR Togolese:ti,ab |  |
| 3 | 'neoplasm'/exp OR Neoplasms:ti,ab OR Neoplasm:ti,ab OR Neoplasia:ti,ab OR Cancer:ti,ab OR Cancers:ti,ab OR cancerous:ti,ab OR Tumor:ti,ab OR Tumors:ti,ab OR Tumour:ti,ab OR Tumours:ti,ab OR carcinoma:ti,ab OR carcinomas:ti,ab OR Oncology:ti,ab OR malignancy:ti,ab OR malignancies:ti,ab OR malignant:ti,ab |  |
| 4 | # 1 AND # 2 AND # 3 | 1358 |
| 5 | # 4 AND [embase]/lim NOT ([embase]/lim AND [medline]/lim) | 728 |
| 6 | # 5 AND ('article'/it OR 'conference paper'/it OR 'conference review'/it OR 'editorial'/it OR 'erratum'/it OR 'letter'/it OR 'note'/it OR 'review'/it OR 'short survey'/it) | 151 |

Database:  APA PsycInfo (EBSCO*host*)

| Set # |  | Results |
| --- | --- | --- |
| 1 | DE "Palliative Care" OR DE "Hospice" OR DE "Holistic Health" OR DE "Spirituality" OR DE "Spiritual Well Being" OR DE "Spiritual Care" OR DE "Psychosocial Factors" OR TI (palliative OR “supportive care” OR hospice OR well-being OR wellbeing OR psychosocial OR spirituality OR spiritual OR spiritualities OR holistic) OR AB (palliative OR “supportive care” OR hospice OR well-being OR wellbeing OR psychosocial OR spirituality OR spiritual OR spiritualities OR holistic) |  |
| 2 | TI (“Sub-Saharan Africa” OR “Subsaharan Africa” OR “Africa South of the Sahara” OR “Central Africa” OR “Central African” OR "eastern Africa" OR "east Africa" OR "east African" OR “Southern Africa” OR “South African” OR “West Africa” OR “West African” OR “Western Africa” OR "British Indian Ocean Territory" OR Burundi OR Umurundi OR Abarundi OR Burundian OR Urundi OR Djibouti OR Djiboutian OR "French Somaliland" OR “French Somali” OR Eritrea OR Eritrean OR Ethiopia OR Ethiopian OR Kenya OR Kenyan OR Rwanda OR Ruanda OR Rwandan OR Somalia OR Somalian OR "South Sudan" OR Sudan OR Sudanese OR Tanzania OR Tanzanian OR Zanzibar OR Zanzibari OR Tanganyika OR Uganda OR Ugandan OR Cameroon OR Cameroonian OR “Central African Republic” OR Chad OR Chadian OR Congo OR Congolese OR “Democratic Republic of the Congo” OR “Equatorial Guinea” OR “Equatorial Guinean” OR Equatoguinean OR Gabon OR Gabonese OR Gabonaise OR “Sao Tome and Principe” OR “Sao Tomean” OR Santomean OR Angola OR Angolan OR Botswana OR Botswanan OR Motswana OR Lesotho OR Lesothan OR Mosotho OR Basotho OR Lesothonian OR Malawi OR Malawian OR Mozambique OR Mozambican OR Namibia OR Namibian OR “South Africa” OR Swaziland OR Swazi OR Zambia OR Zambian OR Zimbabwe OR Zimbabwean OR Benin OR Beninese OR Beninois OR “Burkina Faso” OR Burkinese OR Burkinabe OR “Cabo Verde” OR “Cape Verde” OR “Cabo Verdean” OR “Cape Verdean” OR “Cote d'Ivoire” OR “Ivory Coast” OR Ivorian OR Gambia OR Gambian OR Ghana OR Ghanaian OR Guinea OR Guinean OR “Guinea-Bissau” OR Bissau-Guinean OR Liberia OR Liberian OR Mali OR Malian OR Mauritania OR Mauritanian OR Niger OR Nigerian OR Nigeria OR Senegal OR Senegalese OR “Sierra Leone” OR “Sierra Leonean” OR Togo OR Togolese) OR AB (“Sub-Saharan Africa” OR “Subsaharan Africa” OR “Africa South of the Sahara” OR “Central Africa” OR “Central African” OR "eastern Africa" OR "east Africa" OR "east African" OR “Southern Africa” OR “South African” OR “West Africa” OR “West African” OR “Western Africa” OR "British Indian Ocean Territory" OR Burundi OR Umurundi OR Abarundi OR Burundian OR Urundi OR Djibouti OR Djiboutian OR "French Somaliland" OR “French Somali” OR Eritrea OR Eritrean OR Ethiopia OR Ethiopian OR Kenya OR Kenyan OR Rwanda OR Ruanda OR Rwandan OR Somalia OR Somalian OR "South Sudan" OR Sudan OR Sudanese OR Tanzania OR Tanzanian OR Zanzibar OR Zanzibari OR Tanganyika OR Uganda OR Ugandan OR Cameroon OR Cameroonian OR “Central African Republic” OR Chad OR Chadian OR Congo OR Congolese OR “Democratic Republic of the Congo” OR “Equatorial Guinea” OR “Equatorial Guinean” OR Equatoguinean OR Gabon OR Gabonese OR Gabonaise OR “Sao Tome and Principe” OR “Sao Tomean” OR Santomean OR Angola OR Angolan OR Botswana OR Botswanan OR Motswana OR Lesotho OR Lesothan OR Mosotho OR Basotho OR Lesothonian OR Malawi OR Malawian OR Mozambique OR Mozambican OR Namibia OR Namibian OR “South Africa” OR Swaziland OR Swazi OR Zambia OR Zambian OR Zimbabwe OR Zimbabwean OR Benin OR Beninese OR Beninois OR “Burkina Faso” OR Burkinese OR Burkinabe OR “Cabo Verde” OR “Cape Verde” OR “Cabo Verdean” OR “Cape Verdean” OR “Cote d'Ivoire” OR “Ivory Coast” OR Ivorian OR Gambia OR Gambian OR Ghana OR Ghanaian OR Guinea OR Guinean OR “Guinea-Bissau” OR Bissau-Guinean OR Liberia OR Liberian OR Mali OR Malian OR Mauritania OR Mauritanian OR Niger OR Nigerian OR Nigeria OR Senegal OR Senegalese OR “Sierra Leone” OR “Sierra Leonean” OR Togo OR Togolese) |  |
| 3 | DE "Neoplasms" OR DE "Benign Neoplasms" OR DE "Breast Neoplasms" OR DE "Endocrine Neoplasms" OR DE "Leukemias" OR DE "Melanoma" OR DE "Metastasis" OR DE "Nervous System Neoplasms" OR DE "Terminal Cancer" OR TI (Neoplasms OR Neoplasm OR Neoplasia OR Cancer OR Cancers OR cancerous OR Tumor OR Tumors OR Tumour OR Tumours OR carcinoma OR carcinomas OR Oncology OR malignancy OR malignancies OR malignant) OR AB (Neoplasms OR Neoplasm OR Neoplasia OR Cancer OR Cancers OR cancerous OR Tumor OR Tumors OR Tumour OR Tumours OR carcinoma OR carcinomas OR Oncology OR malignancy OR malignancies OR malignant) |  |
| 4 | # 1 AND # 2 AND # 3 | 72 |

Database:  Scopus

| Set # |  | Results |
| --- | --- | --- |
| 1 | TITLE-ABS-KEY(palliative OR “supportive care” OR hospice OR well-being OR wellbeing OR psychosocial OR spirituality OR spiritual OR spiritualities OR holistic) |  |
| 2 | TITLE-ABS-KEY(“Sub-Saharan Africa” OR “Subsaharan Africa” OR “Africa South of the Sahara” OR “Central Africa” OR “Central African” OR "eastern Africa" OR "east Africa" OR "east African" OR “Southern Africa” OR “South African” OR “West Africa” OR “West African” OR “Western Africa” OR "British Indian Ocean Territory" OR Burundi OR Umurundi OR Abarundi OR Burundian OR Urundi OR Djibouti OR Djiboutian OR "French Somaliland" OR “French Somali” OR Eritrea OR Eritrean OR Ethiopia OR Ethiopian OR Kenya OR Kenyan OR Rwanda OR Ruanda OR Rwandan OR Somalia OR Somalian OR "South Sudan" OR Sudan OR Sudanese OR Tanzania OR Tanzanian OR Zanzibar OR Zanzibari OR Tanganyika OR Uganda OR Ugandan OR Cameroon OR Cameroonian OR “Central African Republic” OR Chad OR Chadian OR Congo OR Congolese OR “Democratic Republic of the Congo” OR “Equatorial Guinea” OR “Equatorial Guinean” OR Equatoguinean OR Gabon OR Gabonese OR Gabonaise OR “Sao Tome and Principe” OR “Sao Tomean” OR Santomean OR Angola OR Angolan OR Botswana OR Botswanan OR Motswana OR Lesotho OR Lesothan OR Mosotho OR Basotho OR Lesothonian OR Malawi OR Malawian OR Mozambique OR Mozambican OR Namibia OR Namibian OR “South Africa” OR Swaziland OR Swazi OR Zambia OR Zambian OR Zimbabwe OR Zimbabwean OR Benin OR Beninese OR Beninois OR “Burkina Faso” OR Burkinese OR Burkinabe OR “Cabo Verde” OR “Cape Verde” OR “Cabo Verdean” OR “Cape Verdean” OR “Cote d'Ivoire” OR “Ivory Coast” OR Ivorian OR Gambia OR Gambian OR Ghana OR Ghanaian OR Guinea OR Guinean OR “Guinea-Bissau” OR Bissau-Guinean OR Liberia OR Liberian OR Mali OR Malian OR Mauritania OR Mauritanian OR Niger OR Nigerian OR Nigeria OR Senegal OR Senegalese OR “Sierra Leone” OR “Sierra Leonean” OR Togo OR Togolese) |  |
| 3 | TITLE-ABS-KEY(Neoplasms OR Neoplasm OR Neoplasia OR Cancer OR Cancers OR cancerous OR Tumor OR Tumors OR Tumour OR Tumours OR carcinoma OR carcinomas OR Oncology OR malignancy OR malignancies OR malignant) |  |
| 4 | # 1 AND # 2 AND # 3 | 879 |
