# Supplementary material for: Quality of life among patients with cancer and their family caregivers in the Sub-Saharan region: A systematic review of quantitative studies
Source: PLOS Glob Public Health. 2022 Mar 31;2(3):e0000098. doi: 10.1371/journal.pgph.0000098 (PMC10021310; doi:10.1371/journal.pgph.0000098)
Supplement: S2 Appendix — (DOCX) [file pgph.0000098.s003.docx]

**Appendix 2: Detailed Quality Assessment of Studies**

| **Author** | **Is the sampling strategy relevant to address the research question?** | **Is the sample representative of the target population?** | **Are the measurements appropriate?** | **Is the risk of nonresponse bias low?** | **Is the statistical analysis appropriate to answer the research question** |
| --- | --- | --- | --- | --- | --- |
| Abebe, 2020 | Y | N | Y | N/A | Y |
| Brown, 2012 | Y | Y | Y | N | Y |
| Cooper, 2001 | Y | Y | Y | N/A | Y |
| Elumelu, 2015 | Y | N | Y | N/A | Y |
| Esan, 2020 | Y | N | Y | N/A | Y |
| Fatiregun, 2017 | Y | Y | Y | Y | Y |
| Gabriel, 2021 | Y | Y | Y | N/A | Y |
| Greeff, 2012 | Y | N | Y | Y | Y |
| Harding, 2011 | Y | N | Y | N/A | Y |
| Ibrahim 2019 | Y | Y | N | Y | Y |
| Kamau 2007 | Y | Y | N/A | N/A | Y |
| Kizza, 2020 | Y | Y | Y | N/A | Y |
| Kugbey 2019* | Y | Y | Y | N/A | Y |
| Kugbey. 2019* | Y | Y | Y | N/A | Y |
| Kyei, 2020 | Y | N | Y | N/A | N |
| Marete, 2010 | Y | N | Y | N/A | N/A |
| Ndetei, 2018 | Y | Y | N/A | Y | Y |
| Ndiok, 2018 | Y | Y | N/A | N/A | Y |
| Ogoncho, 2016 | Y | N | N | N/A | Y |
| Ohaeri, 1998 | Y | N | N/A | N/A | Y |
| O'Hare, 1988 | Y | N | N | N | Y |
| Okoli, 2019 | Y | N | Y | N | Y |
| Onyeneho, 2021 | Y | Y | Y | N/A | Y |
| Ratshikana-Moloko 2020 | Y | Y | N/A | Y | Y |
| Rayne, 2017 | Y | N | N | N/A | Y |
| Wang’ombe, 2021 | Y | Y | Y | Y | Y |
| Yifru,2021 | Y | Y | Y | Y | Y |

*One study with two published reports; Y= Yes; N= No; N/A= Could not determine
