# Supplementary material for: Quality of life among patients with cancer and their family caregivers in the Sub-Saharan region: A systematic review of quantitative studies
Source: PLOS Glob Public Health. 2022 Mar 31;2(3):e0000098. doi: 10.1371/journal.pgph.0000098 (PMC10021310; doi:10.1371/journal.pgph.0000098)
Supplement: Attachment — Submitted filename: Response to Reviewers.docx [file pgph.0000098.s004.docx]

**Response to The Revision Request**

**Date: Nov, 04, 2021**

Re: Resubmission of manuscript “Quality of life among patients with cancer and their family caregivers in the Sub-Saharan Region: a systematic review of quantitative studies" PGPH-D-21-00606

Dear Editors and Reviewers,

Thank you for the opportunity to revise our manuscript*, Quality of life among patients with cancer and their family caregivers in the Sub-Saharan Region: a systematic review of quantitative studies*. It is our belief that the manuscript is substantially improved after making the suggested edits. Following this letter are the Editor/reviewers’ comments and the journal requirements with our responses in *italic* *font*, including how and where the text have been modified. Changes made in the manuscript are marked using highlighted font as requested in the decision letter. The revision has been developed in consultation with the coauthor, and each author has given approval to the final form of this revision. Thank you for your consideration.

Sincerely,

Lixin Song, PhD, RN, FAAN

Associate Professor & Beerstecher-Blackwell Distinguished Scholar

School of Nursing, CB 7460

Lineberger Comprehensive Cancer Center

University of North Carolina-Chapel Hill

Chapel Hill, NC  27599

919.966.3612

[lsong@unc.edu](mailto:lsong@unc.edu)

**RESPONSES**

**The Editor:** Although, I agree that there are variations/inconsistencies in reporting of QOL relate problems in the published literature and estimation of prevalence and severity of QOL problems is not straight forward. However, looking at the tables and reviewing the findings of published literature, I observed quite a few papers have utilized similar tools to estimate QOL among patients and care givers. So, if there is a possibility of making some plots (Forest) for subgroup of studies, which used similar tools and reported scores of same domains (physical, social, spiritual etc.), I would strongly recommend that this type of analysis will strengthen this manuscript as authors are claiming this is a "systematic review of quantitative studies". So rather than just writing that there is variation/heterogeneity/inconsistencies in reported findings of studies, there should be a serious attempt to group some similar studies and run some plots to see effect sizes.

*Thank you for your comments. We agree with you that making some plots (Forest) for subgroup of studies would have strengthened this manuscript. Our findings showed that some studies used the same measurement including FACT-B (n=4), FACT-G (n=3), and EORTC QLQ-C30 (n=3). Unfortunately, these studies have reported QOL domains inconsistently. For example, the studies that used the FACT-B measurements (n=4) have reported psychological domain findings inconsistently, which has made the subgroup analysis and plots (Forest) impossible. Specifically, 1^st^ study reported that Active coping and religious coping were positively correlated with emotional wellbeing; the 2nd study reported low levels of depression (mean: 5.92) and anxiety (mean: 7.51); the 3rd study reported access to health information was negatively correlated with depression and anxiety; and the 4^th^ study only reported low level of emotional wellbeing (mean: 6.98). We observed the same issue with all measurements across all studies. For this reason, we believe that Plot (forest) graph is not applicable.*

**Journal Requirements:**

1. PLOS Global Public Health has specific requirements for systematic reviews and meta-analyses. In order to meet our requirements, please provide:
   a) the page numbers on the PRIMA checklist (File S1) where information can be found in your manuscript.

*The checklist has been completed.*

b) a Supplemental file of the results of the quality assessment for each individual study assessed, reporting the outcome for each individual criteria considered (this would be more detailed than table 4).

*The Supplemental file has been added (S2 Appendix).*

2. Please update the completed 'Competing Interests' statement, including any COIs declared by your co-authors. If you have no competing interests to declare, please state "The authors have declared that no competing interests exist". Otherwise please declare all competing interests beginning with the statement "I have read the journal's policy and the authors of this manuscript have the following competing interests:"

*We have updated the statement.*

3. Please amend your Data Availability Statement and indicate where the data may be found

*We have updated the statement as requested.*

4. State what role the funders took in the study. If the funders had no role in your study, please state: “The funders had no role in study design, data collection and analysis, decision to publish, or preparation of the manuscript.”

*We have updated our statement.*

**Reviewer #1:** The title is good and reflects the context of the systematic review. The abstract is good but however, the introduction section can be improved upon. The methodology is good. From paper selection down to analysis. However, the inclusion criteria can be set to accommodate papers that were published after the WHO framework on QOL among patients was introduced since that is the model that the study is based upon.

*Thank you for your comments. Regarding the introduction, we think that we have included all required parts of the introduction section (i.e., Introduce the topic, describe the background, establish the research problem and the rationale for the review, specify objectives). Regarding the inclusion criteria, we used WHO framework on QOL to synthesize QOL findings. However, we included all studies that have reported QOL findings regardless of their publication date; we hoped to maximize the number of studies from different countries and cancer populations so that our review findings are more generalizable.*

**Reviewer #2:** The inclusion criteria exclude articles without an English full text. Why is this? This may exclude a massive part of the (West-) African population that is French speaking and used to publish in francophone journals. I assume that some of the "ineligible studies excluded (n=1079)" are excluded because of this. The results show no articles from a French speaking country. Of course, I do understand the difficulties of having interpreters/translators within the research team and the methodological hurdles using translated texts. Nevertheless, it must be mentioned as a limitation of the study that your result may not represent SSA as a whole. Before the background of the current discussion around "neocolonialism in Global Health", every researcher needs to be sensitive in the choice of their methodology and must at least discuss around a probable neglecting of local reality through the eyes of "Western researchers" (yes, I acknowledge 3 co-authors from Malawi, however, Malawi belongs to the East-African English-speaking region).

*Thank you for the feedback. We have added this limitation as suggested. “Another major limitation is that the literature search was restricted to English language publications due to the funding and personnel constraints. Therefore, findings of this review only represent those English-speaking countries in the SSA region, which may have largely limited the cross-cultural generalizability of our findings in French speaking countries in the SSA region”.*

**Reviewer #2:** The second point is that the authors do not discuss the already existing "African APCA POS" outcome measurement tool. The authors mention in their recommendations that a "cultural adapted tool" is needed and future research should have a focus on this. Why not discussing around an existing "African-grown" tool that is available in many local languages. I strongly suggest to adapt the discussion chapter around this tool and acknowledge the already existing "culturally appropriate" efforts that have been made in this field.

*Thank you for the feedback. We clarified in the discussion as suggested. “Despite current limitations in assessment tools that we identified the African Palliative Care Association African Palliative Outcome Scale (APCA African POS) has proved to offer a potential validity in measuring QOL for African cancer patients receiving palliative care. The APCA African POS has demonstrated sensitivity to change over time assessed in multiple domains among cancer populations. This tool can be used in future research to provide insight into the related QOL domains, which will enable comparison of QOL among cancer populations in different sociocultural settings in SSA”.*

**Reviewer #2:** Lastly, a minor comment: Lines 302 - 335 are a repetition of the result part. This can be shortened.

*Thank you for your comment. Results were presented in text and tables. Given the large, diverse sets of information in our findings, we briefly summarized the main results to ease readers’ efforts.*
